# Comparison of Patient Outcome Measures between a Traditional Teaching Hospitalist Service and a Non-Teaching Hospitalist Service at an Academic Children’s Hospital

**DOI:** 10.4172/2161-0665.1000336

**Published:** 2017-11-16

**Authors:** Tony R Tarchichi, Jessica Garrison, Kwonho Jeong, Anthony Fabio

**Affiliations:** 1 Department of Pediatrics, Children’s Hospital of Pittsburgh of UPMC, Pittsburgh, USA; 2 Department of Pediatrics, University of Pittsburgh, Pittsburgh, USA

**Keywords:** Pediatric illness, Bronchiolitis, Gastroenteritis

## Abstract

**Background and objectives:**

Inpatient pediatric care is increasingly provided by pediatric hospitalists. This, in addition to changes in resident duty hour restrictions, has led to the creation of new models of care for inpatient pediatric patients. The objective of this study was to compare traditional outcome measures between a pediatric hospitalist-only service and a more traditional academic service in which care was provided by pediatric hospitalists, residents, and medical students. Attending physicians on the hospitalist-only service had an average of 1.7 years of post-residency experience compared to an average 16 years of experience for those working on the traditional academic service.

**Methods:**

This retrospective cohort study (hospitalist-only v. teaching service) used electronic medical records data of patients (n=1,059) admitted to a quaternary care, academic, children’s hospital in Pittsburgh Pennsylvania with diagnoses of bronchiolitis, viral syndrome, and gastroenteritis from July 2011 to June 2014. Primary outcome measures included length of stay, hospital costs, and readmission rates.

**Results:**

Patients with a diagnosis of bronchiolitis admitted to the hospitalist-only service had a significantly higher severity-of-illness-score than those admitted to the teaching service. A decreased length of stay and lower hospital costs were seen for patients admitted to the hospitalist-only service; however, these differences did not reach a level of statistical significance.

**Conclusion:**

There were no statistically significant differences in the outcome measures of patients with common pediatric illnesses admitted to a hospitalist-only versus a teaching hospitalist service. The model of a hospitalist-only service staffed by recent residency graduates may provide an efficient and effective model of care as patients admitted to this service had similar outcome measures to those patients cared for by more-experienced attending physicians.

## Introduction

In the United States, general medical inpatient care is increasingly provided by hospitalists. The growth of pediatric hospital medicine has led to the development of more than thirty pediatric hospital medicine fellowship programs, and in October 2016 the American Board of Medical Specialties recognized pediatric hospital medicine as its own subspecialty [1]. Although the quality and efficiency of inpatient hospitalist care for adults has been studied more extensively than for pediatric patients, prior studies have also demonstrated that inpatient care provided by pediatric hospitalists is associated with lower costs and shorter length of stay [2–5].

Hospitals have employed pediatric hospitalists to provide patient care in a variety of different models. Only a few studies have compared the outcomes of care provided by different pediatric hospitalist system structures [6–8]. A study conducted by Dwight et al. at a tertiary care, academic children’s hospital compared the outcomes of general pediatric patients admitted to two different hospitalist services. Patients were admitted to a non-teaching attending-only service or to a more traditional academic service. There was a significant reduction in length of stay (LOS) for patients admitted to the non-teaching service with no associated increase in mortality or readmission rates [6]. Bekmezian et al. examined a different model of pediatric hospitalist care at another tertiary care, academic children’s hospital. Patients admitted to the gastroenterology and hematology/oncology services were cared for by general pediatricians on a hospitalist-only service or by a traditional academic service overseen by pediatric subspecialty attendings. Patients admitted to the hospitalist-only service had lower hospital costs and shorter LOS [7]. In a study conducted at an academic community hospital, Boyd et al. compared outcomes for patients cared for by a private hospitalist group contracted by local pediatricians to those cared for by an established faculty practice employed by a hospital. They found that patients cared for by the faculty hospitalists had significantly higher severity of illness indices, lower hospital costs and shorter LOS. Medical students and residents were involved in the care of both groups of patients [8].

In general, prior studies have demonstrated that hospitalist-only services can provide care to pediatric patients with shorter LOS and decreased cost. Academic hospitals must balance patient outcome goals with the educational goals of their trainees. The aim of the current study was to compare the effectiveness of different pediatric hospitalist systems in an academic setting. The study was conducted at a quaternary care, academic children’s hospital in Pittsburgh, Pennsylvania. All patients were cared for by pediatric hospitalist attending physicians. One group of patients was admitted to a non-teaching service with twenty-four hour attending coverage. The other group of patients was admitted to a more traditional academic service in which care was provided by twenty-four hour resident coverage and was overseen by teaching hospitalist physicians. The structure of the hospitalist systems in this study is similar to that described by Dwight et al. [6]. However, the amount of clinical service time teaching attendings provided inpatient care was considerably higher for physicians in the current study. The post-residency experience of the pediatric hospitalists was also examined.

## Methods

### The hospitalist team structures

Patients admitted to the general pediatrics service at Children’s Hospital of Pittsburgh are admitted to four different teams, each of which is designated by a color. A patient is assigned to a team based upon the order in which the hospital admissions office begins the process of assigning him or her to a hospital bed. The admission process for general pediatric patients is random and is not influenced by diagnosis, patient demographics, or perceived educational value of the case. One of the four general pediatric teams designated the “orange team,” is a non-teaching service. Attending hospitalist physicians provide in-house coverage of the patients assigned to this team twenty-four hours per day. Approximately 6 months of the year, a senior pediatric resident may rotate on the team during weekdays as an elective experience and is supervised by the orange team attending. No other residents or medical students rotate on this service. The remaining three teaching hospitalist teams are covered by pediatric residents twenty-four hours per day. Medical students also rotate on these teams. Teaching attendings are present in the hospital during the day and are available when not in-house by phone for any resident questions regarding patient care.

### Data collection and analysis

Data for the study were obtained from the Children’s Hospital of Pittsburgh electronic medical records database. The study period was from July 1, 2011 through June 30, 2014. Data from all patients admitted to the general pediatric teams with a diagnosis of bronchiolitis, viral syndrome, or gastroenteritis were included. Demographic characteristics including age, sex, and insurance type (private versus medicaid) were obtained. Primary outcome measures included length of stay (measured in days), readmission rates, and hospital costs. Severity of illness score (SOI) was also calculated based on the All Patient Refined Diagnosis Related Groups (APR-DRG) developed by the National Association of Children’s Hospitals and Related Institutions (NACHRI). This is a nationally used and easily reproducible system which scores severity of illness from 1, representing mild disease, to 4, representing severe disease [9]. T-test or Wilcoxon rank-sum test for continuous variables and chi-square test or Fisher’s exact test for categorical variables were used to compare outcome measures and SOI scores between patients admitted to the orange team (non-teaching service, 24 hour in-house attending coverage) versus those admitted to the more traditional teaching hospitalist teams (24 hour in-house resident coverage).

## Results

### Baseline characteristics

During the study period, 1,059 patients were admitted to the general pediatrics service with diagnoses of bronchitis, viral syndrome, and gastroenteritis. Of these, 190 (18%) were admitted to the orange team and 869 (82%) were admitted to the traditional teaching hospitalist teams. Table 1 demonstrates the baseline characteristics of the patients admitted to these two groups. There was no statistically significant difference in race, sex, or type of insurance. Data were also analyzed based on admitting diagnosis and again no statistically significant differences in the baseline characteristics of the patients admitted to the two different services were seen (Tables 2–4).

### Outcome measures

As demonstrated in Table 1, overall length of stay was 0.5 days shorter for patients admitted to the orange team (3.02 days ± 2.18 compared to 3.52 days ± 5.09). However, this did not reach a level of statistical significance. Hospital costs were higher for those patients admitted to the traditional teaching hospitalist service with an average cost of $ 7330.64 ± 17811.31 versus an average cost of $4995.79 ± 2544.65, but again this difference was not statistically significant. Readmission rates for patients admitted to the two different services were also not significantly different with a rate of 2.1% for the orange team and 1.5% for the traditional teaching hospitalist service.

The severity of illness (SOI) score was significantly higher for patients with bronchiolitis admitted to the orange team as compared to those admitted to the traditional teaching hospitalist service as seen in Table 4. This difference in SOI score was not seen for the diagnoses of viral syndrome or gastroenteritis (Tables 2 and 3). Also, as demonstrated in Tables 2–4, there was no significant difference in the outcome measures of LOS, hospital cost, or readmission rates when data were analyzed based upon patients’ admitting diagnosis.

## Discussion

Prior studies have demonstrated that hospitalist only services can decrease length of stay and hospital costs [6,7]. Although the only outcome measure found to be statistically significant in this study was a higher SOI score for patients with bronchiolitis admitted to the orange team (non-teaching service), this study adds to the body of literature demonstrating the effectiveness of different models of care which can be provided by pediatric hospitalists.

The attending physicians on the orange team were more recent residency graduates with less clinical experience than those attendings who staffed the traditional teaching hospitalist service. At the time of the study, the average number of post-residency years for the orange attendings was 1.7 years compared to 16 years for those attendings on the traditional teaching service. Despite the difference in prior clinical experience, the less experienced orange team attendings were able to provide care with similar outcome measures to those of their more experienced colleagues.

As above, the physicians on the orange team cared for patients with bronchiolitis who were more ill (had higher SOI scores). The 24 hour in-house presence of the orange team attendings likely contributed to their ability to care for patients with bronchiolitis who had higher severity of illness. Although not specifically measured, this may have also prevented some patients with bronchiolitis from being transferred to the ICU, which would reduce overall hospital costs. In addition, although the differences were not statistically significant, the average LOS and hospital costs were lower for patients admitted to the orange team compared to those admitted to the teaching hospitalist service. Therefore, the model of having two different team structures similar to that described in this study may allow recent residency graduates to care for patients on a hospitalist service with less responsibility for educating trainees. Creating hospitalist positions for recent graduates with limited teaching responsibilities may be a cost effective way for hospitals to reduce the clinical responsibilities of their trainees while allowing for similar patient outcomes.

Although it did not reach a level of statistical significance, the average LOS was 0.5 days shorter for those patients admitted to the orange team. In this study, LOS was calculated in days rather than hours and was based upon the time a patient’s nurse recorded that the patient left his or her hospital room rather than the time that a discharge order was placed. The EMR data available at the time of the study did not allow for determination of LOS to be based on the timing of admission and discharge orders. Thus, the LOS data was affected by factors outside physician control including availability of patient transportation from the hospital and availability of nursing staff to review discharge paperwork. These factors were likely similar for both groups of patients and so would be unlikely to lead to differences between the groups.

The admitting diagnoses of bronchiolitis, viral syndrome, and gastroenteritis were chosen as they are common diagnoses allowing for a higher number of patients to be included in the study and to allow for comparison of patients admitted with the same diagnoses to the two different hospitalist groups. Also, patients with these diagnoses are commonly admitted to hospitalist teams throughout the country in both academic and community hospitals. However, the selection of these diagnoses is also a limitation of the study. Given the short length of stay overall for patients with these diagnoses, differences are small and, as discussed above, this study only allowed for measurement of LOS in days rather than hours, making it more difficult to identify a statistically significant difference.

Additional factors that were not studied and could be evaluated in future investigations include measures of patient satisfaction, faculty job satisfaction, and adherence to institutional clinical effectiveness guidelines. These guidelines for the management of common diagnoses are based on relevant literature and expert opinion and are designed to promote evidence-based practice and consistency of care within the hospital. Adherence to such guidelines could be used as a measure of quality of care.

## Conclusion

This study demonstrates different models of care provided by pediatric hospitalists can lead to similar outcome measures. Recent residency graduates with less clinical experience formed a team of pediatric hospitalists on a non-teaching service at a quaternary care pediatric hospital. The outcome measures for their patient, including length of stay and hospital cost, were similar to those of their more experienced colleagues who worked on a more traditional teaching service. The physicians on the non-teaching service also cared for patients with bronchiolitis who had a higher severity of illness score.

## Figures and Tables

**Table 1:** Descriptive statistics for all diagnoses.

All diagnoses	Orange (n=190)	DRG (n=869)	p-value‡
**Age**	0.92 ± 2.64	1.11 ± 3.28	0.4670 t
**Race**			
White	73.68%	75.14%	0.888 c
African American	21.05%	20.25%	
Others/Don’t Know	5.26%	4.60%	
**Sex**			
Male	54.21%	53.97%	0.952 c
Female	45.79%	46.03%	
**SOI**			
1	50.00%	53.62%	0.072 c
2	36.84%	32.91%	
3	12.63%	9.90%	
4	0.53%	3.57%	
**Diagnosis**			
Gastroenteritis	5.79%	3.80%	0.358 c
Viral disease	25.26%	23.36%	
Bronchiolitis	68.95%	72.84%	
**Insurance**			
Medicaid	60.00%	59.95%	0.991 c
Private/commercial	40.00%	40.05%	
**LOS (in days)**	3.02 ± 2.18	3.52 ± 5.09	0.1842 t
**Readmission rates**	2.1% (n=4/190)	1.5% (n=13/869)	0.526
**Total payments**	4995.79+2544.65	7330.64+17811.31	0.0718 t

‡ : t = ttest, c=Pearson’s chi-square test, W=Wilcox rank-sum test, F=Fisher’s exact test

**Table 2:** Descriptive statistics for gastroenteritis.

Gastroenteritis	Orange (n=11)	DRG (n=33)	p-value
**Age**	3.27 ± 5.31	6.18 ± 6.76	0.1762 w
**Race**			
White	54.55%	78.79%	0.245 F
African American	27.27%	15.15%	
Others/Don’t Know	18.18%	6.06%	
**Sex**			
Male	27.27%	54.55%	0.169 F
Female	72.73%	45.45%	
**SOI**			
1	27.27%	27.27%	0.633 F
2	63.64%	42.42%	
3	9.09%	24.24%	
4	0.00%	6.06%	
**Insurance**			
Medicaid	45.45%	63.64%	0.314 F
Private/Commercial	54.55%	36.36%	
**LOS (in days)**	3.09 ± 1.97	3.06 ± 2.30	0.6346 w
**Total payments**	5227.25+951.47	5177.48+2369.94	0.3209 w

**Table 3:** Descriptive statistics for viral disease.

Viral Disease	Orange (n=48)	DRG (n=203)	p-value
**Age**	2.29 ± 4.07	3.04 ± 5.23	0.3525 t
**Race**			
White	72.92%	79.31%	0.277 F
African American	22.92%	19.21%	
Others/Don’t Know	4.17%	1.48%	
**Sex**			
Male	50.00%	50.25%	0.976 c
Female	50.00%	49.75%	
**SOI**			
1	64.58%	54.19%	0.395 F
2	25.00%	34.48%	
3	8.33%	10.34%	
4	2.08%	0.99%	
**Insurance**			
Medicaid	60.42%	62.56%	0.783 C
Private/commercial	39.58%	37.44%	
**LOS (in days)**	2.17 ± 1.36	2.19 ± 1.36	0.9249 t
**Total payments**	4847.57+1781.66	5031.59+3017.96	0.6853 t

**Table 4:** Descriptive statistics for bronchiolitis.

Bronchiolitis	Orange (n=131)	DRG (n=633)	p-value
**Age**	0.22 ± 0.59	0.22 ± 0.58	0.9744 t
**Race**			
White	75.57%	73.62%	0.864 c
African American	19.85%	20.85%	
Others/Don’t Know	4.58%	5.53%	
**Sex**			
Male	58.02%	55.13%	0.546 c
Female	41.98%	44.87%	
**SOI**			
1	46.56%	54.82%	0.008** c
2	38.93%	31.91%	
3	14.50%	9.00%	
4	0.00%	4.27%	
**Insurance**			
Medicaid	61.07%	58.93%	0.650 c
Private/commercial	38.93%	41.07%	
**LOS (in days)**	3.32 ± 2.37	3.97 ± 5.83	0.2127 t
**Total payments**	5030.66+2860.57	8180.1+20732.80	0.0833 t
